# Feasibility of app-based pulmonary telerehabilitation program for textile dyeing workers with respiratory symptoms: a quasi-experimental study

**DOI:** 10.12701/jyms.2026.43.20

**Published:** 2026-03-02

**Authors:** Myeong Geun Jeong, Gun Seo Jung, Kyoung Tae Kim

**Affiliations:** Department of Rehabilitation Medicine, Keimyung University Dongsan Hospital, Keimyung University School of Medicine, Daegu, Korea

**Keywords:** Breathing exercises, Mobile applications, Occupational diseases, Respiratory disease, Telerehabilitation

## Abstract

**Background:**

Textile dyeing workers are at an increased risk of developing occupational respiratory diseases owing to frequent exposure to harmful chemicals, irritants, and high humidity in the workplace. Pulmonary rehabilitation is essential but associated with access and adherence challenges. This study aimed to evaluate the feasibility, effectiveness, accessibility, and safety of an app-based pulmonary telerehabilitation program for this population.

**Methods:**

Forty-five textile dyeing workers with respiratory symptoms underwent a 4-week pulmonary telerehabilitation program consisting of daily gamified respiratory muscle training using a Bluetooth-enabled device and video-guided flexibility exercises. The program was delivered through a mobile application and adherence was monitored using an online platform. Pulmonary function tests, 6-minute walk test, hand grip strength, and St. George’s Respiratory Questionnaire (SGRQ) scores were assessed at baseline (T0) and post-intervention (T1). The adherence, adverse events, and system usability were evaluated.

**Results:**

Improvements were observed in forced expiratory volume in 1 second (FEV1), FEV1/forced vital capacity, maximal inspiratory pressure, and maximal expiratory pressure at T1 compared to T0. The SGRQ activity scores improved after the intervention. The mean adherence rate was 95.69%; three participants had adherence rates <80% for ≥1 week each. The mean System Usability Scale score was high.

**Conclusion:**

The program resulted in significant improvements in pulmonary function and quality of life with high adherence rates and good usability. This may be a promising approach for managing occupational respiratory diseases among textile dyeing workers. Further studies with larger sample sizes and longer follow-up periods are warranted to validate these results and assess the long-term outcomes.

## Introduction

Chronic respiratory diseases (CRDs) are globally prevalent noncommunicable diseases primarily associated with widespread exposure to harmful environmental, occupational, and lifestyle-related inhalants [1]. CRDs encompass a range of conditions that affect the lungs and airways, including chronic obstructive pulmonary disease (COPD), asthma, pneumoconiosis, and interstitial lung disease. In 2019, CRDs were identified as the third leading cause of mortality worldwide [2]. The economic burden of healthcare associated with CRDs has been steadily increasing worldwide [3].

Industrial areas are particularly susceptible to CRDs [4]. Workers in the textile dyeing industry are frequently exposed to toxic allergens, irritants, and high humidity. These toxins can cause acute symptoms, including headache, itchy and irritated eyes, and chronic respiratory symptoms such as cough, dyspnea, phlegm, and chest tightness [5,6]. Furthermore, prolonged exposure is associated with the development of respiratory diseases, including COPD, occupational asthma, respiratory infections, and pulmonary fibrosis [7,8]. Occupational respiratory diseases impose a high economic burden, necessitating regular health checkups, early symptom detection, self-care activities, and workplace environmental monitoring [9,10]. Proper diagnosis and treatment, including pulmonary rehabilitation (PR), are required [11].

Recent emphasis in PR has been placed on lung function, and physical and exercise capabilities to overcome respiratory symptoms. Decreased respiratory function can result in compromised physical activity, muscle weakness, and deconditioning, significantly affecting daily tasks and the overall quality of life (QoL) [12, 13]. Therefore, PR is essential for maintaining respiratory and physical functions. It involves physical exercises, breathing retraining, respiratory muscle strengthening, flexibility training, and psychological and nutritional support [14]. However, PR has low adherence rates owing to fear of breathlessness, increased symptoms of exacerbation, transportation issues, and hospitalization [15,16]. In addition, advanced age and frailty have been shown to curb the adoption of rehabilitation programs.

Several solutions have been proposed to overcome these barriers [17]. Digital therapeutics such as mobile applications (i.e., apps) for COPD self-management and telerehabilitation for various respiratory diseases have been developed and evaluated [18]. Telerehabilitation is more cost-effective than face-to-face rehabilitation and exhibits positive results in terms of feasibility, safety, and effectiveness [19,20]. However, it also faces challenges such as a lack of digital literacy and skills required for eHealth, and concerns related to data privacy [21]. Nevertheless, recent technological advancements have led to telerehabilitation being strongly recommended with moderate-quality evidence in the American Thoracic Society 2023 guidelines [22].

This study aimed to evaluate the implementation of app-based pulmonary telerehabilitation for textile dyeing workers with respiratory symptoms. The intervention included exergames featuring respiratory muscle training and video-guided flexibility exercises. The program’s feasibility, effectiveness, accessibility, and safety were assessed.

## Methods

**Ethics statement:** This study was approved by the Institutional Review Board (IRB) of Keimyung University Dongsan Hospital (IRB noNo: DSMC 2023-07-056) and written informed consent was obtained from all participants. This study was also registered in the Clinical Research Information Service (CRIS) Registry (Registration Number: KCT0008793).

### 1. Patient recruitment and selection

Textile dyeing workers with respiratory symptoms were recruited from the Daegu Dyeing Industrial Complex (DDIC) between September 1, 2023 and October 31, 2023. The complex comprised 126 companies, and most workers were primarily engaged in the dyeing and finishing of textile fabrics, power plants, and shared wastewater treatment facilities. Participants who signed a consent form after receiving a thorough explanation of the study and met the inclusion criteria were enrolled. The inclusion criteria were DDIC workers presenting with respiratory symptoms, including dyspnea, cough, sputum, and chest pain. The exclusion criteria were as follows: (1) inability to undergo functional assessment or use a respiratory training device, (2) pregnancy or presence of severe illnesses in addition to CRD, (3) patients diagnosed with and undergoing treatment for respiratory diseases before study enrollment, and (4) inability to utilize apps or execute the treatment program because of cognitive impairment or lack of digital literacy.

### 2. Outcome measures

The participants underwent comprehensive functional assessments and completed a patient report questionnaire pre-intervention (T0) and post-intervention (T1). Pulmonary function tests were conducted, including forced vital capacity (FVC), forced expiratory volume in 1 second (FEV1), maximal inspiratory pressure (MIP), maximal expiratory pressure (MEP), and peak expiratory flow using Pony FX (COSMED Inc., Rome, Italy) and Peak Flow Meter (Clement Clarke International, Mountain Ash, UK). The 6-minute walk test (6MWT) and hand grip strength (HGS) test were performed to evaluate physical function. The St. George’s Respiratory Questionnaire (SGRQ) was used to evaluate QoL.

At T1, system usability [23], participant satisfaction, and adverse events were assessed using a questionnaire designed to evaluate treatment sustainability. The system usability scale (SUS), developed to evaluate the overall usability of the app-based telerehabilitation software, consisted of 10 items rated on a 5-point Likert scale ranging from 1 (strongly disagree) to 5 (strongly agree), yielding a total score from 0 to 100, with higher scores indicating better usability. The participant satisfaction questionnaire consisted of 24 items rated on a 5-point Likert scale ranging from 1 (strongly disagree) to 5 (strongly agree), yielding a total score from 24 to 120. The questionnaire was developed based on relevant literature and the authors’ previous experiences (Supplementary Material 1). The adverse events questionnaire was specifically developed to evaluate two domains: (1) changes in respiratory symptoms following the intervention, and (2) common adverse effects associated with respiratory training, including dizziness and hyperventilation-related symptoms (Supplementary Material 2).

### 3. Intervention

**Fig. 1.**
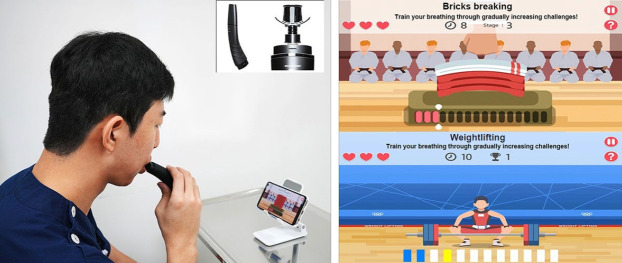
Mobile app-based pulmonary telerehabilitation using the Breath-On device and embedded exergame.

The pulmonary telerehabilitation program consisted of gamified respiratory muscle training and flexibility exercises. Respiratory muscle training was conducted using the Breathe-On system (HUWANT Corp., Daegu, Korea) (Fig. 1). When inhalation and exhalation are performed through a mouthpiece, the load cell detects pressure changes within the closed space, recognizes the inhalation and exhalation epochs, and quantifies their intensity and duration. The app, linked to the Breathe-On device by Bluetooth, provides gamified respiratory muscle training.

The participants performed one daily session consisting of two types of exergames, repeated sequentially, 5 days per week. Each exergame was conducted in three sets with five trials per set. Five flexibility exercises were performed in each set. During the 4-week intervention period, the first 2 weeks were set at a lower intensity to allow the participants to adapt to the treatment. The intensity gradually increased during weeks 3 and 4. Although adverse events were evaluated at T1, the participants were asked to report any unusual symptoms or side effects during the intervention, including dizziness or headache. No specific restrictions or guidelines were imposed on the participants’ lifestyles or physical activities.

On the same day, after the initial evaluation (T0), the researchers provided the participants with a Breathe-On device and installed the app on their smartphones. Owing to the self-administered nature of the telerehabilitation program, participants were required to perform the exercises independently without direct supervision. Therefore, several strategies were implemented to mitigate the risk of data loss and dropouts, which may have arisen from poor adherence, operational inexperience, and system errors. Upon enrollment, the researcher explained the operational protocol and provided a comprehensive demonstration in a stepwise manner. Subsequently, participants were supervised to ensure that they could conduct the intervention independently.

To enhance adherence to the intervention, daily pop-up notifications for respiratory training were automatically displayed on the participant’s smartphone. In addition, data from the completed training sessions were recorded and transmitted to an online administrative platform to evaluate the adherence of each participant. If the training completion rate fell below 80% in a week or if the participant did not train for 2 consecutive days, reminders were sent via phone calls to encourage participation.

### 4. Exergame for respiratory muscle training

The respiratory muscle training exergame included “Brick-breaking” and “Weightlifting.” It operated by interacting with the inhalation and exhalation of each participant, providing reciprocal feedback.

In “Brick-breaking,” the physiologic mechanism of pursed lip breathing (PLB) was adopted, which is known to be an effective breathing technique for patients with various pulmonary diseases. The participants were required to maintain consistent exhalation according to the duration (in seconds) displayed on the screen while the expiratory pressure was set to 15 cmH_2_O [24]. If exhalation was maintained for the target duration, the gauge on the screen was filled, and the digital avatar simulated the breaking of a tile. If constant exhalation was not maintained, it was considered a failure and there was no tile-breaking animation [25].

Each trial lasted 15 seconds, with a mandatory 10-second resting period between trials. The completion of five trials constituted one set. In weeks 3 and 4, the target duration of exhalation gradually increased from 2 seconds in weeks 1 and 2 to 4 and 6 seconds in weeks 3 and 4, respectively.

In “Weightlifting,” an additional pre-inhalation step (pressure resistance, 10 cmH_2_O) was added, followed by exhaling. When the target expiratory pressure was achieved, a digital avatar simulated lifting a weight. The exercise was considered successful when the displayed gauge was filled during inhalation and then completely filled during exhalation. The other intervention protocol was the same as that for “Brick-breaking.” The pressure of exhalation resistance gradually increased from 20 cmH_2_O in weeks 1 and 2 to 44 and 68 cmH_2_O in weeks 3 and 4, respectively.

### 5. Flexibility exercises

Between each set of “Brick-breaking” and “Weightlifting,” the participants were prompted to perform a 1-minute flexibility exercise by following a video guide with detailed instructions displayed in the app: shoulder rolling, upward and forward chest stretching, chest relaxation, and side stretching.

### 6. Statistical analysis

Descriptive statistics were used to analyze demographics and post-intervention patient-reported questionnaires. The paired *t*-test was used for the comparative analysis of outcomes before and after pulmonary telerehabilitation. To examine whether demographic factors influenced treatment responses, sensitivity analyses were performed using analysis of covariance with age and sex as covariates. Adherence to pulmonary telerehabilitation was assessed as a percentage by calculating the number of days on which app-based respiratory training sessions were performed out of the total number of planned training days. Statistical analyses were performed using IBM SPSS version 21.0 (IBM Corp., Armonk, NY, USA). Statistical significance was set at *p*<0.05.

## Results

A total of 45 textile dyeing workers in the DDIC (42% female) with a mean age of 46.96±10.0 years were enrolled. Coughing was the most common symptom, with a mean modified Medical Research Council score of 0.4±0.49; dyspnea was not severe (Table 1). All participants completed 4 weeks of pulmonary telerehabilitation.

**Fig. 2.**
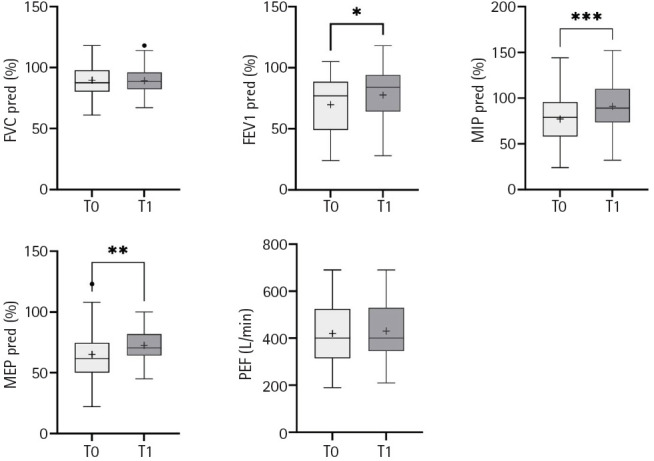
Temporal changes in pulmonary function test between baseline (T0) and post-intervention (T1). FVC, forced vital capacity; pred, predicted; FEV1, forced expiratory volume in 1 second; MIP, maximal inspiratory pressure; MEP, maximal expiratory pressure; PEF, peak expiratory flow. Dots indicate outliers. *p<0.05, **p<0.01, ***p<0.001.

**Fig. 3.**
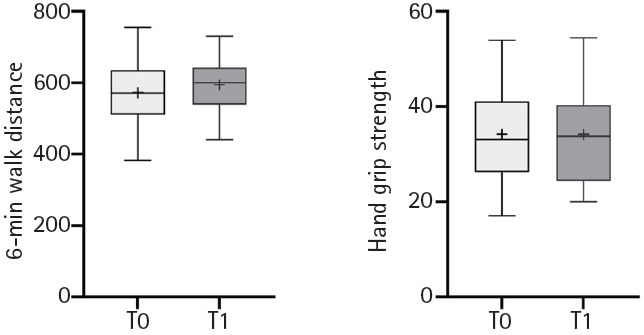
Temporal changes in physical performance between baseline (T0) and post-intervention (T1).

**Fig. 4.**
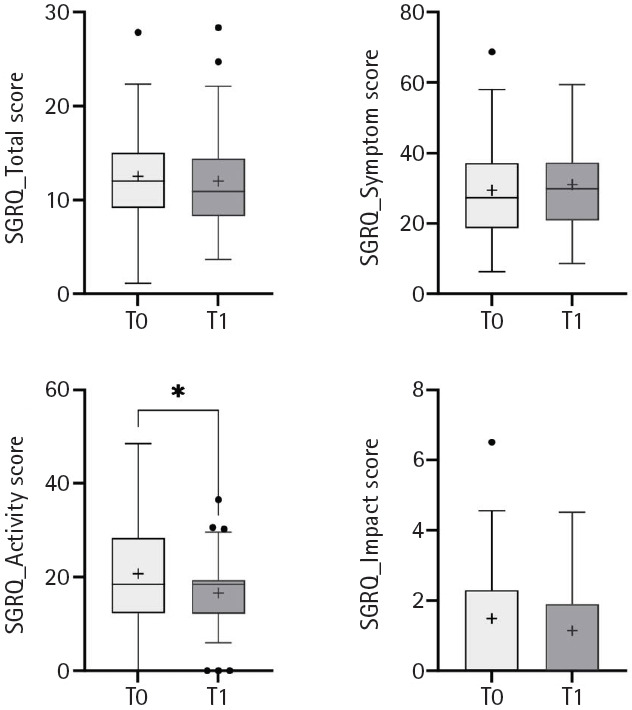
Temporal changes in quality of life between baseline (T0) and post-intervention (T1). SGRQ, St. George’s Respiratory Questionnaire. Dots indicate outliers. *p<0.05.

Pulmonary function significantly improved in terms of FEV1, FEV1/FVC, MIP, and MEP at T1 (Table 2, Fig. 2). The 6MWT improved, although the difference was not statistically significant (Table 2, Fig. 3). A significant improvement was observed in the activity portion of the SGRQ (Table 2, Fig. 4). After adjusting for age and sex, results remained consistent. Neither age nor sex significantly modified treatment effects (all *p* for interaction >0.05), and covariates explained minimal variance (R² <0.1 for most models) (Supplementary Table 1).

The mean SUS score was 71.39±13.86, which is considered acceptable with an “OK” usability level (Table 3) [23]. The mean satisfaction score was 98.8±2.72 out of 120. Most items received scores close to the average of 4 points. However, the item “Would you be willing to purchase the Breathe-On at your own expense?” received the lowest score, with a mean of 2.93±1.05 (Supplementary Table 2). No significant adverse events were observed during the study period. The mean score of adverse events was 7.24±1.98 out of 30 and the lowest score was 6. A detailed analysis of the questionnaire responses revealed that the item “Did you experience shortness of breath during the respiratory training?” received the highest score with a mean value of 1.67±0.95 (Supplementary Table 3).

Notably, no reminder phone calls were made to encourage treatment, and adherence was 95% with automated pop-up notifications alone.

## Discussion

Textile dyeing workers with respiratory symptoms underwent a 4-week pulmonary telerehabilitation program using a smartphone exergame with an integrated respiratory muscle training device. Participation resulted in significant improvements in pulmonary function and QoL, without any major adverse events. No dropouts occurred during the intervention, and the overall adherence rate was 95%.

The textile dyeing industry, which involves processes such as dyeing, bleaching, and finishing, poses a high risk of exposure to volatile organic compound (VOC) emissions [26,27]. VOCs are hazardous air pollutants that can cause immediate and chronic health problems. Toluene, xylene, and chloroform are the main VOCs, which often lead to neurological problems and damage to internal organs.

Reactive dyes cause occupational respiratory diseases in dyeing workers [28]. Docker et al. [29] found that >15% of workers handling reactive dyes had respiratory symptoms attributed to irritation from chemicals, such as hydrochloric acid vapors, sulfur dioxide, and the dyes themselves. These exposures can lead not only to respiratory symptoms but also to the development of conditions such as COPD, chronic cough, dyspnea, and pulmonary fibrosis due to the inhalation of dust particles. Additionally, the mean percentage of predicted forced expiratory flow was significantly lower in the exposed workers (25%) than in the unexposed workers (75%).

Established in 1980, the DDIC is instrumental in regional industrial development. Despite its diminished scale owing to the decline in related industries, it remains a prominent dyeing industrial complex in South Korea that employs many workers. This complex encompasses various facilities in addition to primary dyeing operations, including power plants and wastewater treatment facilities. Shuai et al. [26] found that the incidence of respiratory diseases, including bronchiolitis and asthma, in the exposed area of the DDIC was significantly higher than that in a controlled area located 10 km away [30].

The present study employed pulmonary telerehabilitation comprising relatively simple elements such as respiratory muscle training and flexibility exercises. Workers with respiratory symptoms but without a prior diagnosis of respiratory disease were enrolled.

Several studies have been conducted on remote or telerehabilitation devices for patients with COPD. Tey et al. [30] established a remote PR system using motion sensing data in which patients wore headbands and used a personal computer-based rehabilitation application for motion tracking. Broadbent et al. [31] proposed a remote rehabilitation approach using a robot that conducted rehabilitation treatment at home for 4 months; 75% of the patients responded that the robot was helpful for medication, education, and companionship. However, these interventions have limitations compared to Breathe-On training, as they rely on personal computers or have restrictions owing to the poor mobility of robots. Breathe-On, in contrast, has the advantages of being small, portable, and lightweight, allowing for greater usability, and it is engaged via a smartphone-linked breathing exergame.

The exergame consists of “Brick-breaking” and “Weightlifting,” which mimic inspiratory and expiratory muscle training. It facilitates the diaphragm and deep abdominal muscles [32] to enhance coughing, sputum discharge, and stable breathing [33]. The PLB mechanism used in exergames maintains positive end-expiratory pressure by creating back pressure in the airways. This opens the airway wider, prevents the early collapse of small bronchioles, improves overall lung function, and stabilizes breathing [25]. The 1-minute flexibility exercise between training sets was also essential to improve lung function. Additionally, a regular resting period alleviates fatigue, dizziness, and dyspnea that can occur during breathing exercises [34].

Respiratory muscle training can elicit physiological alterations that resemble those observed in hyperventilation owing to prolonged respiratory cycle duration and increased tidal volume [35,36]. Hyperventilation induces hypocapnia and acidosis, which causes vasoconstriction of many organs [37]. In particular, hyperventilation may reduce cerebral blood flow, leading to headaches, dizziness, and fainting in severe cases. These hyperventilation-like symptoms are commonly observed during inspiratory and expiratory muscle training.

To prevent dizziness commonly associated with frequent over-breathing while using Breathe-On, parameters such as the frequency of respiration and target pressure were adjusted [38], and videos demonstrating flexibility exercises were inserted between each training set to minimize adverse effects. The low incidence of adverse events associated with telehealth may have contributed to decreased adherence [39]. Therefore, in this study, extensive efforts were made to ensure that the related symptoms did not occur during respiratory training.

During the 4-week PR training, the average adherence rate was 95.69%, which may be attributed to the use of gamified training content to engage the participants in telerehabilitation [40]. Participation was also facilitated by the compact size and user-friendly nature of the Breathe-On system. To further enhance adherence, pop-up reminders encouraging daily participation in breathing exercises were displayed on the app, and only three participants showed adherence rates <80% for at least 1 week. Usage instructions were provided before the intervention and the researchers provided thorough explanations through demonstrations to improve understanding.

The mean satisfaction score for pulmonary telerehabilitation was 98.8 out of 120. When reviewing the survey items, the users gave low scores to their “willingness to purchase the device at their own expense.” Participants reported frequent sensor malfunctions caused by moisture and poor wireless connections, suggesting that errors during training discourage users from spending money on the device, and that the device needs to be more user-friendly and requires a higher level of technical refinement for commercialization. However, this error did not compromise intervention adherence.

In this study, MIP and MEP significantly improved after pulmonary telerehabilitation, with minimal clinically important differences [41]. Aktan et al. [42] evaluated telerehabilitation through video calls at home and found that the MIP and MIP% predicted significant improvements.

However, the 6MWT and HGS scores did not show any statistically significant improvement. It may be assumed that the intervention protocol in this study did not include aerobic or resistance training; therefore, the potential to improve physical function was lacking.

The high adherence rate (95.69%) and acceptable usability score (71.39) demonstrated the commercial viability of the Breathe-On device. However, several factors must be addressed for successful commercialization. First, technical refinement is required to resolve sensor malfunctions caused by moisture and connectivity issues, as identified in our satisfaction survey. Second, cost-effectiveness should be considered, because the participants were reluctant to purchase the device at their own expense (mean score, 2.93), suggesting the need for insurance coverage or employer-sponsored programs for occupational health management.

This study has several limitations. First, the small sample size limited the generalizability of our findings. In addition, as this study included all workers with respiratory symptoms, future research should focus on patients diagnosed with specific respiratory diseases to enhance the applicability of the results. Second, the 4-week duration of the rehabilitation program may have been insufficient to fully assess improvements in pulmonary and physical functions. Long-term follow-up after the cessation of treatment is required to determine any residual effects. Third, the absence of aerobic exercise and lifestyle modifications in the intervention protocol may have limited the comprehensive assessment of the PR effectiveness. Eventually, the target resistance pressures for inspiration and expiration training were uniform for healthy adults. To maximize the treatment effect, the individualized target pressure should be based on the initial MIP and MEP assessments. However, in this study, a uniform pressure threshold was provided owing to technical limitations. Future improvements in the device should allow personalized respiratory training.

This study demonstrated the effectiveness of app-based pulmonary telerehabilitation in textile dyeing workers with respiratory symptoms. Breathe-On delivered significant improvements in pulmonary function including FEV1, FEV1/FVC, MEP, and MIP. Improvements in QoL were also reported in the SGRQ assessment. No substantial usability issues were encountered with the mobile device application, and the participants showed remarkably high adherence to telerehabilitation. Individuals can perform PR at home without spatial constraints using the device, and the gamified format may lead to high adherence and sustained treatment participation. A future pulmonary telerehabilitation program that includes physical exercise would be advantageous in facilitating treatment.

## Figures and Tables

**Table 1.** Participants’ demographic characteristics

Characteristic	Data
No. of patients	45
Age (yr)	46.96±10.00
Body mass index (kg/m^2^)	23.92±3.73
Sex	
Male	26 (57.8)
Female	19 (42.2)
Symptoms	
Coughing	39 (86.7)
Sputum	1 (2.2)
Chest discomfort	9 (20.0)
Smoker	15 (33.3)
mMRC	0.40±0.49

Values are presented as number (%) or mean±standard deviation. mMRC, modified Medical Research Council.

**Table 2.** Comparison of pulmonary function, physical performance, and quality of life at pre-intervention (T0) and post-intervention (T1)

Variable	T0	T1	*p*-value
Pulmonary function			
FVC (L)	3.57±0.86	3.59±0.93	0.79
FVC (% pred)	89.68±12.73	89.20±11.68	0.80
FEV1 (L)	2.22±0.87	2.50±0.87	0.02*
FEV1 (% pred)	69.84±23.95	77.73±21.62	0.04*
FEV1/FVC	0.62±0.20	0.69±0.16	0.03*
MIP (cmH_2_O)	71.82±29.18	84.04±28.68	0.001*
MEP (cmH_2_O)	72.49±23.20	82.40±18.85	<0.001*
PEF (L/min)	420.1±133.40	431.1±126.50	0.43
Physical performance			
6MWT (m)	571.70±95.56	593.90±69.10	0.05
HGS (kg)	34.17±10.43	34.20±10.15	0.96
SGRQ	12.52±5.13	12.00±5.31	0.45
Symptoms	29.43±14.39	31.03±11.97	0.38
Activity	20.75±12.54	16.64±8.29	0.01*
Impacts	1.49±1.93	1.14±1.51	0.39

Values are presented as mean±standard deviation. FVC, forced vital capacity; FEV1, forced expiratory volume in 1 second; MIP, maximal inspiratory pressure; MEP, maximal expiratory pressure; PEF, peak expiratory flow; 6MWT, 6-minute walk test; HGS, hand grip strength; SGRQ, St. George’s Respiratory Questionnaire.

* Statistical significance at *p*<0.05.

**Table 3.** Patient-reported outcome and adherence to pulmonary telerehabilitation

Variable	T1
Satisfaction score	98.80±12.72 (total, 120)
Adverse events score	7.24±1.98 (total, 6–30)
System usability scale	71.39±13.86 (total, 100)
Adherence (%)	95.69±5.48

Values are presented with mean±standard deviation. T1, post-intervention.

## Supplementary materials

Supplementary Material 1. User satisfaction questionnaire

Supplementary Material 2. Adverse events questionnaire

Supplementary Table 1. ANCOVA results for mean difference after adjusting for age and sex

Supplementary Table 2. Result of user satisfaction evaluation

Supplementary Table 3. Result of adverse events evaluation
