# Supplementary material for: Feasibility of app-based pulmonary telerehabilitation program for textile dyeing workers with respiratory symptoms: a quasi-experimental study
Source: J Yeungnam Med Sci. 2026 Mar 2;43:20. doi: 10.12701/jyms.2026.43.20 (PMC13107087; doi:10.12701/jyms.2026.43.20)
Supplement: Supplementary Material 1. — User satisfaction questionnaire [file jyms-2026-43-20-Supplementary-Material-1.pdf]

Supplementary Material 1. User satisfaction questionnaire

• Please read the following statements and circle the number that best represents your level of satisfaction

No.	Item	Highly dissatisfied	Dissatisfied	Neutral	Satisfied	Highly satisfied
1	Were you satisfied with the design of the device in terms of usability?	1	2	3	4	5
2	Was the overall use of the device convenient?	1	2	3	4	5
3	Were the device instructions sufficiently clear?	1	2	3	4	5
4	Was the device's grip comfortable enough to use?	1	2	3	4	5
5	Was the Bluetooth pairing process between the device and your smart device convenient?	1	2	3	4	5
6	Were the application buttons easy to understand and use?	1	2	3	4	5
7	How actively did you participate in the respiratory training?	1	2	3	4	5
8	Was the explanation of the respiratory training easy to understand?	1	2	3	4	5
9	Was the difficulty level of the respiratory training appropriate?	1	2	3	4	5
10	How well do you think your breathing was reflected in the device during the respiratory training?	1	2	3	4	5
11	Was the respiratory training convenient to perform without causing fatigue?	1	2	3	4	5
12	How much enjoyment did you feel during the respiratory training compared to other exercises?	1	2	3	4	5
13	Compared to other exercises, how helpful was the respiratory training in maintaining consistent?	1	2	3	4	5
14	How effective do you think the respiratory training was compared to other exercises?	1	2	3	4	5
15	Do you think the respiratory training will help improve your respiratory function?	1	2	3	4	5
16	Would you recommend the respiratory training to others?	1	2	3	4	5
17	Do you think the respiratory training would be highly useful if done at home?	1	2	3	4	5
18	Would you be willing to purchase the Breathe-On at your own expense?	1	2	3	4	5
19	What is your overall satisfaction score for the respiratory training?	1	2	3	4	5
20	Was the flexibility exercise easy to follow?	1	2	3	4	5
21	Was the intensity of the flexibility exercise appropriate?	1	2	3	4	5
22	Did the flexibility exercise allow for sufficient rest during the breathing training?	1	2	3	4	5
23	Do you think the flexibility exercise was physically effective?	1	2	3	4	5
24	Was the flexibility exercise helpful in performing the respiratory training?	1	2	3	4	5
				Total score: ______		
