# Supplementary material for: Feasibility of app-based pulmonary telerehabilitation program for textile dyeing workers with respiratory symptoms: a quasi-experimental study
Source: J Yeungnam Med Sci. 2026 Mar 2;43:20. doi: 10.12701/jyms.2026.43.20 (PMC13107087; doi:10.12701/jyms.2026.43.20)
Supplement: Supplementary Material 2. — Adverse events questionnaire [file jyms-2026-43-20-Supplementary-Material-2.pdf]

Supplementary Material 2. Adverse events questionnaire

• Please read the following statements and circle the number that best represents your level of agreement

No.	Item	Strongly disagree				Strongly agree
1	Did you experience shortness of breath during the respiratory training?	1	2	3	4	5
2	Did you experience nausea or dizziness during the respiratory training?	1	2	3	4	5
3	Did you experience other respiratory symptoms (e.g., cough, phlegm, etc.) during the respiratory training?	1	2	3	4	5
4	Did you feel fatigue after the respiratory training?	1	2	3	4	5
5	Did your existing respiratory symptoms (cough, phlegm, difficulty breathing, etc.) worsen after the respiratory training?	1	2	3	4	5
6	Did you experience any severe adverse events that required a hospital visit during or after the respiratory training?	1	2	3	4	5
				Total score: ______		

* If you experienced any discomfort during the respiratory training, please describe the reasons below:

____
