# Supplementary material for: Feasibility of app-based pulmonary telerehabilitation program for textile dyeing workers with respiratory symptoms: a quasi-experimental study
Source: J Yeungnam Med Sci. 2026 Mar 2;43:20. doi: 10.12701/jyms.2026.43.20 (PMC13107087; doi:10.12701/jyms.2026.43.20)
Supplement: Supplementary Table 1. — ANCOVA results for mean difference after adjusting for age and sex [file jyms-2026-43-20-Supplementary-Table-1.pdf]

Supplementary Table 1. ANCOVA results for mean difference after adjusting for age and sex

Variable	Adjusted *p*-value[a)]	Covariate	F	*p*-value	R^2	Adjusted R^2
FVC (L)	0.836					
		Age	0.636	0.430	0.017	−0.030
		Sex	0.127	0.723		
FVC (% predicted)	0.996					
		Age	1.053	0.311	0.026	−0.021
		Sex	0.086	0.771		
FEV1 (L)	0.104					
		Age	2.039	0.161	0.061	0.170
		Sex	0.833	0.367		
FEV1 (% predicted)	0.229					
		Age	0.901	0.348	0.024	−0.230
		Sex	0.151	0.699		
FEV1/FVC	0.295					
		Age	0.006	0.938	0.064	0.020
		Sex	2.860	0.098		
MIP (cmH_2O)	0.099					
		Age	0.889	0.351	0.044	−0.003
		Sex	0.892	0.350		
MEP (cmH_2O)	0.936					
		Age	0.000	0.991	0.012	−0.037
		Sex	0.475	0.495		
PEF (L/min)	0.842					
		Age	0.046	0.831	0.027	−0.020
		Sex	1.112	0.298		
6MWT (m)	0.716					
		Age	0.005	0.945	0.028	−0.019
		Sex	1.190	0.282		
HGS (kg)	0.158					
		Age	2.604	0.114	0.063	0.017
		Sex	0.205	0.653		
SGRQ						
Total score	0.290					
		Age	0.682	0.414	0.130	0.087
		Sex	5.200	0.058		
Symptoms	0.708					
		Age	0.002	0.969	0.024	−0.024
		Sex	1.012	0.320		
Activity	0.907					
		Age	0.104	0.749	0.050	0.004
		Sex	2.010	0.164		
Impacts	0.126					
		Age	1.462	0.234	0.111	0.068
		Sex	3.403	0.072		

FVC, forced vital capacity; FEV1, forced expiratory volume in 1 second; MIP, maximal inspiratory pressure; MEP, maximal expiratory pressure; PEF, peak expiratory flow; 6MWT, 6-minute walk test; HGS, hand grip strength; SGRQ, St. George's Respiratory Questionnaire.
[a)]Adjusted *p*-value: *p*-value from age- and sex-adjusted analysis of covariance (ANCOVA) models.
Statistical significance at $p < 0.05$.
