# Supplementary material for: Feasibility of app-based pulmonary telerehabilitation program for textile dyeing workers with respiratory symptoms: a quasi-experimental study
Source: J Yeungnam Med Sci. 2026 Mar 2;43:20. doi: 10.12701/jyms.2026.43.20 (PMC13107087; doi:10.12701/jyms.2026.43.20)
Supplement: Supplementary Table 2. — Result of user satisfaction evaluation [file jyms-2026-43-20-Supplementary-Table-2.pdf]

Supplementary Table 2. Result of user satisfaction evaluation

Item no.	Mean ± SD
1	4.02 ± 0.81
2	3.93 ± 0.96
3	4.38 ± 0.68
4	4.29 ± 0.79
5	4.18 ± 0.91
6	4.36 ± 0.68
7	4.53 ± 0.63
8	4.56 ± 0.62
9	4.31 ± 0.70
10	4.00 ± 0.80
11	4.38 ± 0.65
12	3.93 ± 0.89
13	3.96 ± 0.90
14	3.69 ± 0.79
15	3.76 ± 0.80
16	3.87 ± 0.89
17	3.98 ± 0.89
18	2.93 ± 1.05
19	3.78 ± 0.97
20	4.42 ± 0.66
21	4.27 ± 0.69
22	4.40 ± 0.69
23	4.02 ± 0.75
24	4.11 ± 0.78

SD, standard deviation.
