# Supplementary material for: Feasibility of app-based pulmonary telerehabilitation program for textile dyeing workers with respiratory symptoms: a quasi-experimental study
Source: J Yeungnam Med Sci. 2026 Mar 2;43:20. doi: 10.12701/jyms.2026.43.20 (PMC13107087; doi:10.12701/jyms.2026.43.20)
Supplement: Supplementary Table 3. — Result of adverse events evaluation [file jyms-2026-43-20-Supplementary-Table-3.pdf]

Supplementary Table 3. Result of adverse events evaluation

Item no.	Mean ± SD
1	1.67 ± 0.85
2	1.31 ± 0.60
3	1.20 ± 0.59
4	1.11 ± 0.32
5	1.02 ± 0.15
6	1.00 ± 0.00

SD, standard deviation.
